# Current morbimortality and one-year survival after pneumonectomy for infectious diseases

**DOI:** 10.1016/j.clinsp.2023.100169

**Published:** 2023-02-14

**Authors:** Paula Duarte D'Ambrosio, Alessandro Wasum Mariani, Eserval Rocha Júnior, Israel Lopes de Medeiros, Leonardo César Silva Oliveira, Antero Gomes Neto, Ricardo Mingarini Terra, Paulo Manuel Pêgo-Fernandes

**Affiliations:** a Instituto do Coração, Hospital das Clínicas, Faculdade de Medicina, Universidade de São Paulo (HCFMUSP), São Paulo, SP, Brazil; b Instituto do Câncer, Hospital das Clínicas, Faculdade de Medicina, Universidade de São Paulo (HCFMUSP), São Paulo, SP, Brazil; c Hospital do Coração de Messejana, Fortaleza, CE, Brazil

**Keywords:** Pneumonectomy, Infectious disease, Tuberculosis, survival, mortality

## Abstract

**Objective:**

Identify the one-year survival rate and major complications in patients submitted to pneumonectomy for infectious disease.

**Methods:**

Retrospective data from all cases of infectious disease pneumonectomy over the past 10 years were collected from two reference centers. The authors analyzed: patient demographics, etiology, laterality, bronchial stump treatment, presence of previous pulmonary resection, postoperative complications in the first 30 days, the treatment used in pleural complications, and one-year survival rate.

**Results:**

56 procedures were performed. The average age was 44 years, with female predominance (55%). 29 cases were operated on the left side (51%) and the most frequent etiology was post-tuberculosis (51.8%). The overall incidence of complications was 28.6% and the most common was empyema (19.2%). Among empyema cases, 36.3% required pleurostomy, 27.3% required pleuroscopy and 36.3% underwent thoracoplasty for treatment. Bronchial stump fistula was observed in 10.7% of cases. From all cases, 16.1% were completion pneumonectomies and 62.5% of these had some complication, a significantly higher incidence than patients without previous surgery (p = 0.0187). 30-day in-hospital mortality was (7.1%) with 52 cases (92.9%) and 1-year survival. The causes of death were massive postoperative bleeding (1 case) and sepsis (3 cases).

**Conclusions:**

Pneumonectomy for benign disease is a high-risk procedure performed for a variety of indications. While morbidity is often significant, once the perioperative risk has passed, the one-year survival rate can be very satisfying in selected patients with benign disease.

## Highlights

• Pneumonectomy for benign disease is a high-risk procedure performed for a variety of indications.
• Patients with benign conditions would be expected to have superior long-term survival in comparison with those with malignancy.
• Morbidity is often significant, with a meticulous operative technique and detailed patient management, which can be similar to a pneumonectomy performed for malignancy.
• Pneumonectomy for a benign disease should continue to be considered a treatment option for carefully selected patients.

## Introduction

Pneumonectomy has frequently been used for the treatment of central bronchogenic carcinoma. Under certain conditions, pneumonectomy is required to address benign lung disease, for carefully selected patients. Indications usually include complications of pulmonary Tuberculosis (TB), cystic bronchiectasis, suppurative lung disease, that immunosuppression combined with opportunistic invasive infection, and fibrosing mediastinitis.^1^

Pneumonectomies for benign diseases are technically challenging procedures. Thoracic surgeons must perform in the setting of dense scar tissue and inflammatory adhesion around major vascular structures. These challenges are combined with a high rate of complications.^1^ The Society of Thoracic Surgeons General Thoracic Surgery Database (STS GTDB) reports that patients undergoing pneumonectomy for benign conditions are at almost three times the risk of major perioperative events when compared with lung cancer patients.^2^ Others, however, advocate that pneumonectomy for benign disease can be performed with a favorable morbidity and mortality range from 23% to 63% and 0% to 25%, respectively.^1^^,^^3^^,^^4^

To comprehensively select patients who would benefit from this surgical procedure, many authors have retrospectively published their experiences to identify risk factors associated with poor outcomes.^1^^,^^4^^,^^5^ However, little data are reported regarding the one-year survival and the long-term outcome following pneumonectomy for benign disease.

In this retrospective analysis, 56 patients with benign lung disease who underwent pneumonectomy from two centers were included. The authors evaluate surgical outcomes of the last ten years to identify the one-year survival rate and major complications.

## Materials and methods

This was a retrospective study performed at two-reference centers: Instituto do Coração (Incor), Hospital das Clínicas HCFMUSP, Faculdade de Medicina, Universidade de São Paulo, and Hospital Messejana, Fortaleza. The study was approved by the Institutional Review Board (CAPPesq HCFMUSP ‒ 33365720.2.0000.0068). The authors retrospectively reviewed the electronic medical records from two thoracic surgery databases who had undergone pneumonectomy for an infectious disease during a 10-year period. The study population consisted of all patients receiving pneumonectomy for infectious diseases. Inclusion criteria: patients undergoing pneumonectomy for infectious disease. There were no exclusion criteria in the current study. Patients who had already undergone some previous ipsilateral pulmonary resections were classified as completion pneumonectomy.

### Data collection

Basic demographic information, including age, sex, comorbidities, smoking history, and etiology of infectious lung disease. Variables collected include surgical technique, bronchial stump treatment, presence of previous pulmonary resection, postoperative complications in the first 30 days, and one-year survival rate.

### Surgical procedure

The procedure was performed in a standard operating room under sterile conditions. Patients underwent general anesthesia with a double-lumen endotracheal tube allowing for selective single-lung ventilation. Surgery was performed in the lateral decubitus position. The surgical procedure was performed by thoracotomy or Videothoracoscopy (VATS), decided by the surgeon. Additionally, the authors did not perform a pleuropneumonectomy, in some cases a greater pleural detachment was necessary, but this does not constitute an extrapleural pneumonectomy. One chest tube was placed after the operation and the chest tube was retained for 24 hours and kept clamped unless mediastinal adjustment of space decompression was required. The chest tube was removed gradually after the fluid collection was below 100 mL per day.

The technique of the pneumonectomy of each participating site was very similar, and in both places, this high-risk procedure was performed for an experienced thoracic surgical procedure without significant changes over the years. Moreover, both centers are reference centers for the treatment of chronic infectious diseases, one in the northeast region and another in the southeast region. However, as they are high-volume hospitals and referral centers, both have training programs for thoracic surgery. Despite having residents participating in the surgical procedure, they were always supervised and performed by the same experienced assistant team during the study period.

### Statistical analysis

Descriptive statistical analysis was used to summarize the characteristics of the studied patients and surgical procedures. Frequencies and percentages are presented for categorical variables, and continuous variables are summarized as the median. A comparison of risk groups was made by using *χ*2 analysis or a non-parametric Wilcoxon test, where appropriate. The Kaplan-Meier method was used to calculate the one-year survival rate. All statistical analyses were performed using the software SPSS (IBM Corp) version 20.0. Probability (p) values of less than 0.05 were considered statically significant.

## Results

56 procedures were performed in the study, of which 32 were women 55%. The mean age was 44 years. Twenty-six patients (68%) had associated comorbidities and smoking history was the most prevalent in thirteen patients (34.2%). Patients’ baseline demographic characteristics and comorbidities are summarized in (Table 1). The most frequent etiologies were post-TB (51.8%), pulmonary aspergillosis (21.4%), bronchiectasis (19.6%), and nontuberculous mycobacteria (3.6%). 29 cases were operated on the left side (51.8%), and only one case was performed by VATS. No intraoperative complications were observed. The bronchial stump was closed by an absorbable manual suture in 44 patients (78.6%), by a stapler in 10 (17.8%), and non-absorbable suture in just two patients.

**Table 1:** Characteristics of study group.

Variables	
Sex, n (%)	
Female	32 (55%)
Male	24 (45%)
Comorbidities, n (%)	
Hipertension	5 (13.2%)
Diabetes Mellitus	3 (7.9%)
Previous neoplasia	2 (5.2%)
Smoking-history	13 (34.2%)
Side of surgical procedure, n (%)	
Right	27 (48.2%)
Left	29 (51%)
Completion pneumonectomy, n (%)	9 (16%)
Post tuberculosis bronchiectasis	29 (51.8%)
Aspergilloma	12 (21.4%)
Bronchiectasis	11 (19.6%)
Invasive Pulmonary Aspergillosis	2 (3.6%)
Nontuberculous mycobacteria	2 (3.6%)
Bronchial stump treatment, n (%)	
Stapler	10 (17.8%)
Absorbable manual	44 (78.6%)
Non-absorble manual	2 (3.6%)
n = 56 patients	

The overall incidence of major complications was 28.6% and the most common were empyema 11 (19.2%) followed by postoperative bleeding 3 (5.2%) (Table 2). Among the 11 patients who developed empyema, all of them underwent a surgical procedure (pleurostomy in 4, decortication in 3, and thoracoplasty in 4). Among the 3 cases that had postoperative bleeding, 1 patient who was reoperated died due to this acute bleeding. In contrast, the other 2 patients were managed clinically with no further interventions.

**Table 2:** Major complications of the study group.

Variables, n (%)	n	(%)
Empyema	11	19.2
Postoperative bleeding	3	5.2
Pneumonia	2	3.5
Death^a^	1^a^	1.7
None	40	70.2
n = 56 patients/ 57 major complications		

a The patient who died, died due to bleeding so the same is found in post-operative bleeding and death.

Bronchial Stump Fistula (BSF) was observed in 6 cases (10.7%) and 16.6% were identified in the first 14 days. In five cases, the bronchial closure technique was by absorbable manual suture and in just one case bronchus was closed by an automatic stapler. From all cases, 8 (16%) were completion pneumonectomies and 62.5% of these had empyema, which assign from this group a significantly higher incidence than patients without previous surgery (p = 0.0187). From these patients with completion pneumonectomies and empyema, the BSP was present in 5 cases (83%).

The one-year survival was 92.9% with only 04 deaths: two due to sepsis related to postoperative empyema, another due to immediate postoperative bleeding, and another due to an undetermined cause several months after hospital discharge.

## Discussion

The destroyed lung is more prevalent in developing countries,^6^ and those patients are at risk of developing fatal complications like massive hemoptysis, aspergillosis, septicemia, and empyema.^7^ It was found that pneumonectomy is an effective method for preventing and treating such serious complications.^8^^,^^9^ The present series of 56 patients undergoing elective pneumonectomy for a variety of chronic infectious diseases. TB was the most common etiology of infectious disease followed by pulmonary aspergillosis in the present study. Despite the high rate of major complications, the one-year survival rate was 94% and compares favorably with modern-day cancer pneumonectomy rates.^5^^,^^10^^,^^11^

Given the complexities of these operations, the most important postoperative complications reported after pneumonectomy included Bronchopleural Fistula (BPF), empyema, reoperation for bleeding, and respiratory failure.^12^ In this study, the overall incidence of major complications was 28.6% and the most common were empyema (19.2%) followed by postoperative bleeding (5.2%). The authors achieved satisfactory surgical outcomes in all patients, except one who died due to intense postoperative bleeding that in the re-operation was identified to be from a rupture of the Pulmonary Artery (PA). The final pathology of this case showed his on the PA wall. The authors don't know if this condition could be related to the PA rupture. Published studies found that the presence of preoperative empyema, aspergillosis, massive blood loss, right pneumonectomy, reoperation for bleeding, and intraoperative dissemination were the most critical issues that lead to significant complications.^12^^,^^13^

A few authors have suggested that patients undergoing completion pneumonectomy are not at an increased risk of complications.^1^^,^^14^ In contrast, a more recent review of outcomes after completion of pneumonectomy noted that patients undergoing completion pneumonectomy for benign conditions were more likely to have major complications and lower median survival than those with malignancy.^13^ The present study did not find higher mortality in the completion pneumonectomy subset, however, completion pneumonectomy was more likely to have major complications such as empyema (p = 0.0187) and BPF.

Some of the best results reported for benign pneumonectomy have come from areas where pulmonary TB is endemic, and thus experience with the operative management of these patients is vast.^15^ In this study, TB was the most common etiology of infectious disease, and the mortality rate was 7.1% for the entire cohort. In a study by Klapper and colleagues on the outcome of pneumonectomy for benign disease, they reported a 29% mortality rate for the entire cohort, and 83% of deaths were operated on an emergent or urgent bias.^16^ They did not report cases of TB.^16^ It has been found that mortality was reduced to less than 10% after the exclusion of the emergency cases,^17^ which is following the present results and other reports.^17^

Although there are a few papers that have reported long-term survival rates after pneumonectomy for chronic inflammatory lung disease, the first-year survival rates in this series would seem to be satisfactory (92.9%). For high-risk patients with preoperative empyema, Shiraishi et al.^18^ reported a 5-year survival rate of 83% and Ashour et al.^19^ reported no long-term death during 12–124 months of follow-up periods after 24 pneumonectomies for TB. In contrast, Speicher^20^ recently reported rewarding long-term results after pneumonectomy for stage I–II NSCLC: they found that 5-year survival rates were 50%, 40%, 29%, and 19% in patients aged 50, 50–69, 70–79, and 79 years, respectively. Certainly, once the perioperative risk has passed, patients with benign conditions would be expected to have superior long-term survival in comparison with those with malignancy. Also, when the pneumonectomy is proposed for inflammatory disease in most cases the surgery is removing an already destroyed lung, in contrast for cancer, where the lung is most of the cases physiologically active, this difference can partially explain the higher incidence of complications due the accommodation in the postoperative period like the cardiac arrhythmia for lung cancer patients^21^ compared to the present data.

The adequate preoperative workup for pulmonary resections in lung infectious diseases is still in debate since the current algorithms validated for lung cancer do not correct postoperative complications.^22^ In this population, it is important to look not only for the cardiac and pulmonary assessment risk but for other details like the nutritional status and possible airway contamination. Additionally, whenever the surgery is indicated for adjuvant treatment for TB (MDR/XDR), chronic cavitary aspergillosis, or non-tuberculosis mycobacteria a correct and rigorous medical treatment is mandatory during the perioperative period. The WHO guidelines for resistant TB, are based on a previously published meta-analysis.^23^ state that the indication for adjuvant lung resection must be done if the surgery is lobectomy or less, due to the higher pneumonectomy mortality. However, recent data contrast this information, and several authors believe that for good candidates pneumonectomy can be a safe and effective adjuvant treatment for TB, leading to good results.^24^

The present study has several limitations, most inherent to the methodology. The main limitation of this study is its retrospective nature, which may have introduced selection biases. The small size is a similar limitation, which makes drawing definitive conclusions difficult. But to the authors’ knowledge, the size of this series compares favorably with other published series and is notable for the spectrum of disease processes that it includes.

## Conclusion

Pneumonectomy for benign lung disease is a high-risk procedure that is performed for a variety of indications. While morbidity is often significant, once the perioperative risk has passed, the one-year survival rate can be very satisfying in this varied patient population. Pneumonectomy for a benign disease should continue to be considered a treatment option in selected patients.

## Funding

This research did not receive any specific grant from funding agencies in the public, commercial, or not-for-profit sectors.

## Footnote

The authors are accountable for all aspects of the work in ensuring that questions related to the accuracy or integrity of any part of the work are appropriately investigated and resolved.

## Declaration of Competing Interest

The authors declare no conflicts of interest.
